# The COVID-19 lockdown promotes changes in sleep habits in the Croatian general population

**DOI:** 10.3325/cmj.2022.63.352

**Published:** 2022-08

**Authors:** Renata Pecotić, Ivana Pavlinac Dodig, Linda Lušić Kalcina, Sijana Demirović, Katarina Madirazza, Maja Valić, Zoran Đogaš

**Affiliations:** 1 Split Sleep Medicine Center, University of Split School of Medicine, Split, Croatia; 2 Department of Neuroscience, University of Split School of Medicine, Split, Croatia; 3 Department of Neuroscience, University of Mostar School of Medicine, Mostar, Bosnia and Herzegovina

## Abstract

**Aim:**

To investigate the effects of the coronavirus disease 2019 (COVID-19) lockdown on sleep habits in the Croatian general population.

**Methods:**

In this cross-sectional study, 1173 respondents from the general population (809 women) completed a self-report online questionnaire that gathered demographic data and data on sleep habits and mood changes before and during the COVID-19 lockdown.

**Results:**

During the lockdown, bedtime (from 23:11 ± 1:07 to 23:49 ± 1:32 h, *P* < 0.001) and waketime were delayed (from 6:51 ± 1:09 to 7:49 ± 1:40 h, *P* < 0.001). Sleep latency increased from 10 (5-20) to 15 (10-30) minutes (*P* < 0.001). Bedtime and waketime delays were more pronounced in women and respondents younger than 30. Compared with other age groups, respondents younger than 30 more frequently reported insomnia for the first time during the lockdown and had less frequent night-time awakenings (*P* < 0.001), less common problems falling asleep (*P* < 0.001), less frequently felt calm (*P* < 0.001) and rested (*P* < 0.001), but more frequently felt sadness (*P* < 0.001) and fear (*P* = 0.028).

**Conclusion:**

The effect of the lockdown on sleep needs to be better understood. Sleep hygiene education could serve a first-line lifestyle intervention for people in lockdown experiencing sleep disruption.

Coronavirus disease 2019 (COVID-19) is a new communicable disease with devastating global effects. Its emergence and spread have caused confusion, anxiety, and fear among the general public (1). In Croatia, the first COVID-19 patient was recorded on February 26, 2020. The lockdown was announced on March 19 and lasted until April 27, when a gradual relaxation of restrictions began (2,3). During that period, the epidemic situation in Croatia was relatively favorable, with 2.1 deaths per 100 000 population (according to EDC Europe).

Populations exposed to restrictive measures, especially those related to the COVID-19 pandemic, suffer from negative psychological, emotional, social, and general health consequences (4-13). The increase in anxiety, stress, and depression during the lockdown might disrupt sleep quality and sleep hygiene (14,15). Furthermore, the balance between circadian rhythm and homeostatic sleep pressure might be affected by environmental factors. For example, excessive use of social media before bedtime, especially in the younger population, negatively affects sleep habits and sleep quality, and increases the risk of sleep disorders (16). As stress-related sleep problems might adversely influence health, and as there is a bidirectional relation between emotional and behavioral reactivity and sleep quality (14,17), the effects of lockdown on sleep disturbances need to be assessed. Sleep hygiene and sleep quality are growing public health issues globally, and their importance has been especially evident during the COVID-19 pandemic. Poor sleep tremendously affects human health as it may lead to reduced cognitive and psychomotor performance, emotional impairment, increased fatigue, sympathetic nervous system activation, and blood pressure rise, cortisol secretion alterations, and development of insulin resistance (18,19). Furthermore, high-quality sleep stimulates the immune system, which emphasizes the role of good sleep habits in decreasing the risk for COVID-19 infection (20-22). Therefore, overall sleep quality and sleep habits are important considerations in people who self-isolate or are in lockdown.

Although the epidemic situation during the COVID-19 lockdown in Croatia was very well controlled, strict measures and fear might have affected sleep, behavior, and mood. Considering that evidence from different populations supports the negative effects of such measures on sleep in general (23-27), this study aimed to assess the effects of the COVID-19 lockdown on sleep habits and the development of sleep disorders, especially insomnia, in the Croatian general population. During the lockdown, respondents from Zagreb experienced an earthquake of magnitude 5.5 on the Richter scale, which might have been a confounding factor.

## Respondents and methods

### Respondents

The study enrolled 1173 respondents (809 or 73.7% women). All respondents were adults from the general population of Croatia who had internet access. The exclusion criterion was age younger than 18.

### Methods

This cross-sectional, online, questionnaire-based study was carried out from April 25, 2020 to May 5, 2020. The questionnaire was distributed to the investigators' contacts through snowball sampling by e-mails and social media (WhatsApp, Viber, Facebook, and others). The investigators' contacts were asked to share the survey with as many people as possible and to encourage their contacts to share the link further. After accepting to participate in the survey, the respondents were automatically directed to the questionnaire by clicking on the link. Because of the distribution and sampling technique, we were not able to calculate the response rate. The study protocol was approved by the Biomedical Research Ethics Committee of the University of Split, School of Medicine. All study procedures conformed to the ethical principles of the 1964 Declaration of Helsinki and its later amendments.

The online, self-report questionnaire was developed by the investigators using Google forms. The questionnaire included a brief explanation of the study aims and procedures, and three subsets of questions: personal and demographic data (17 questions), sleep habits (26 questions), and mood changes (16 questions) before and during the COVID-19 lockdown in Croatia (28). The demographic data section inquired about the respondents' general characteristics and background (birth, sex, height, weight, current residence, education, and employment status). The sleep habits section inquired about bedtime and waketime, sleep latency, naps, awakenings during the night, difficulties while falling asleep, self-reported insomnia, chronic fatigue, and other sleep-related problems such as snoring, sleep-walking, bruxism, and sleep-talking before and during the lockdown. The total sleep time was calculated as the difference between bedtime and waketime. Sleep latency was self-reported as a response to an open-ended question, expressed in minutes. The sleep habits questionnaire had been previously used in studies published by our group (16,28,29). In the mood changes sections, the respondents self-assessed calmness, rest, contentment, anxiety, anger, fear, discouragement, and sadness before and during the lockdown on a Likert scale from 1 to 4 (1 – not at all, 2 – somewhat, 3 – moderately, 4 – very much so).

### Statistical analysis

The normality of distribution was assessed with a Kolmogorov-Smirnov test and Shapiro-Wilks test. The homogeneity of variances was also assessed. When a deviation from normality was found, parametric analysis was performed if visual inspection following the assessment of the Q-Q plots and distribution plots revealed a distribution close to normal. We assumed that type-I error might be adequately controlled even in skewed populations when having a large sample size. If the same assessment revealed asymmetric data along with a significant test of normality, a Wilcoxon signed-rank test for skewed variables was used for paired samples to assess the change between before and during the COVID-19 lockdown. A *t* test for paired samples was performed when possible according to underlying assumptions. An analysis of variance (ANOVA) was performed to compare continuous variables in different age groups. When homogeneity of variances assumption was violated, a Brown-Forsythe and Welch parameter was calculated, since this parameter is not based on the assumption that all the groups were sampled from populations with equal variances. Differences among specific groups were tested with a *post-hoc* Tukey test. Logistic regression included categorical and continuous variables as predictors. When categorical variables were included in the logistic regression, the first category was used as the reference category. Statistical significance level was set at *P* < 0.05.The statistical analysis was performed with SPSS, version 14 (IBM, Armonk, NY, USA).

## Results

Overall, 73.7% of respondents were female, and the average age of the total sample was 42 years (Table 1). The majority of respondents had a master’s degree (49.4%) or PhD (12.5%). Overall, 95.6% of respondents were adhering to the lockdown restrictions most of the time or all the time, with the majority of them working from home (41.9%) or in rotating shifts (15.4%).

**Table 1:** Respondents’ demographic characteristics

Characteristics	N (%)
**Distribution**	
female	809 (73.7)
male	288 (26.3)
**Age (median, IQR)**	42 (32-52)
male	46 (35-55)
female	42 (31-52)
**Body weight (median, IQR)**	72 (63-84)
**Height (median, IQR)**	173 (168-179)
**Body mass index (median, IQR)**	23.94 (21.71-26.93)
**Education**	
elementary school	9 (1)
high school	206 (23.4)
college or bachelor’s degree	121 (13.7)
master’s degree	435 (49.4)
PhD	110 (12.5)
**Restrictions**	
I don't follow restrictions	5 (0.4)
I follow restrictions occasionally	46 (3.9)
I follow restrictions most of the time	473 (40.3)
I follow all the restrictions	649 (55.3)
**Smoking**	
no	850 (72.5)
yes	323 (27.5)

### Shift in sleep patterns during the COVID-19 lockdown

Bedtime was delayed from 23:11 ± 1:07 hours before the lockdown to 23:49 ± 1:32 hours during the lockdown (P*<*0.001). Waketime was also delayed from 6:51 ± 1:09 hours before the lockdown to 7:49 ± 1:40 hours during the lockdown (P*<*0.001), with the delay being even than that for bedtime. Sleep latency was increased from 10 (5-20) minutes before the lockdown to 15 (10-30) minutes during the lockdown (P*<*0.001), with a similar increase in women and men (Table 2).

**Table 2:** Sleep latency before and during the lockdown in both sexes. Data are presented as median (interquartile range)

	Sleep latency (min)		
	before lockdown	during lockdown	P*
**Total sample**	10 (5-20)	15 (10-30)	<0.001
**Male**	10 (5-15)	15 (7.5-30)	<0.001
**Female**	15 (5-30)	20 (10-30)	<0.001

*Wilcoxon signed-rank test.

Bedtime and waketime delay was more pronounced in women compared with men (Table 3) and in patients under 30 compared with other age groups. On the other hand, the delay was similar in all age-groups older than 50. Middle-aged population, aged from 30 to 50 years, mostly reported a somewhat smaller bedtime and waketime delay when compared with younger respondents (Table 3).

**Table 3:** Sex differences in change in bedtime and waketime during lockdown

	Mean ± standard deviation	P*
**Change in bedtime during the lockdown (bedtime after-bedtime before)**		0.029
female	0:40 ± 1:21	
male	0:28 ± 1:12	
**Change in waketime during the lockdown (waketime after-waketime before)**		
female	0:59 ± 1:26	0.032
male	0:47 ± 1:21	
**Change in bedtime during lockdown (bedtime after-bedtime before)**		
up to 29 years^†^	1:00 ± 1:01	<0.001
30-39 years^‡^	0:37 ± 1:17	
40-49 years^‡^	0:39 ± 1:04	
50-59 years^§^	0:21 ± 1:04	
above 60 years^¶^	0:11 ± 1:04	
**Change in waketime during lockdown (waketime after-waketime before)**		
up to 29 years^†^	1:34 ± 1:11	<0.001
30-39 years**	1:00 ± 1:20	
40-49 years^‡^	0:55 ± 1:14	
50-59 years^††^	0:36 ± 1:17	
above 60 years^¶^	0:25 ± 1:10	

*T-test for equality of means, equal variances not assumed and ANOVA test was performed. †Significantly different to all other age groups (post-hoc Tukey test).

‡Significantly different to age group up to 29 years and above 60 years (post-hoc Tukey test).

§Significantly different to age group up to 29 years (post-hoc Tukey test).

¶Significantly different to age groups up to 29 years, 30-39 years, 40-49 years (post-hoc Tukey test).

**Significantly different to age group up to 29 years, 50-59 years, and above 60 years (post-hoc Tukey test).

††Significantly different to age group up to 29 years and 30-39 years (post-hoc Tukey test).

Respondents from Zagreb, who experienced an earthquake during the lockdown, did not differ from other respondents in the frequency of night-time awakenings (χ ^2^ = 0.433; *P* = 0.511) and difficulties falling asleep (χ^2^ = 0.213; *P* = 0.644) before the lockdown. However, during the lockdown they reported more night-time awakenings (χ^2^ = 13.405; P*<*0.001) and difficulties falling asleep (χ^2^ = 8.644; *P* = 0.003). They did not report a significantly different shift in bedtime (*P* = 0.673) and waketime (*P* = 0.134) compared with other respondents.

### Newly recognized insomnia patients during the COVID-19 lockdown

Overall, 169 (15.4%) respondents reported having insomnia before the lockdown, whereas 316 (28.8%) reported it during the lockdown. Therefore, 220 respondents during the lockdown self-reported insomnia for the first time. These respondents were younger than other respondents (39 [28.5-48] years vs 43 [33-54] years; P*<*0.001). Sixty-one respondents self-reported insomnia before but not during the lockdown.

**Figure 1:**
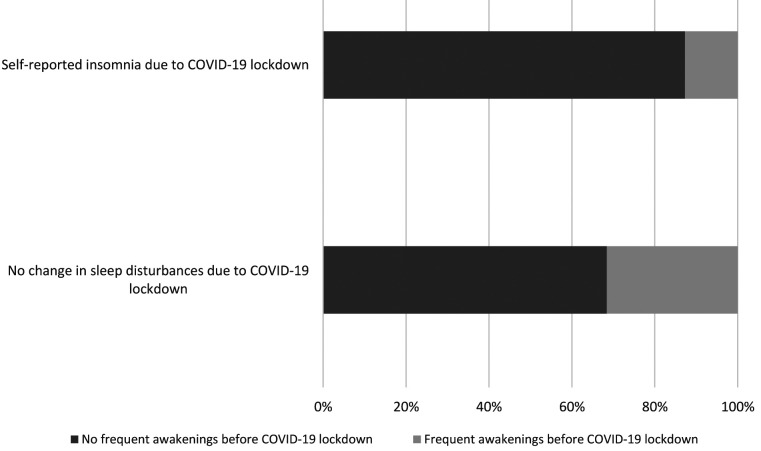
The percentage of respondents with self-reported insomnia and those without sleep disturbances due to the coronavirus disease 2019 (COVID-19) lockdown among respondents with and without frequent awakenings before the lockdown.

**Figure 2:**
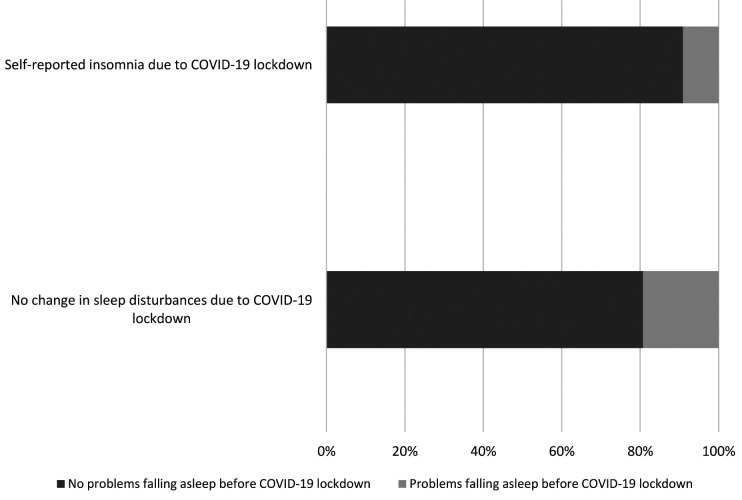
The percentage of respondents with self-reported insomnia and those without sleep disturbances due to the coronavirus disease 2019 (COVID-19) lockdown among respondents with and without problems falling asleep before the lockdown.

Respondents with newly reported insomnia reported less frequent awakenings (12.7% vs 31.6%, P*<*0.001) and less common problems falling asleep before the lockdown (9.1% vs 19.3%, P*<*0.001) compared with respondents with no change in sleep disturbances (Figures 1 and 2). They did not report having other chronic diseases more often (19.1%) than respondents who reported no change in sleep disturbances (22.4%). Respondents with newly recognized insomnia did not significantly differ from other respondents according to sex (χ^2^ = 2.84, *P* = 0.092), education (χ^2^ = 6.49, *P* = 0.166), domestic living conditions (χ^2^ = 5.59, P *=* 0.349), or working status (χ^2^ = 4.67, P*<*0.198).

**Figure 3:**
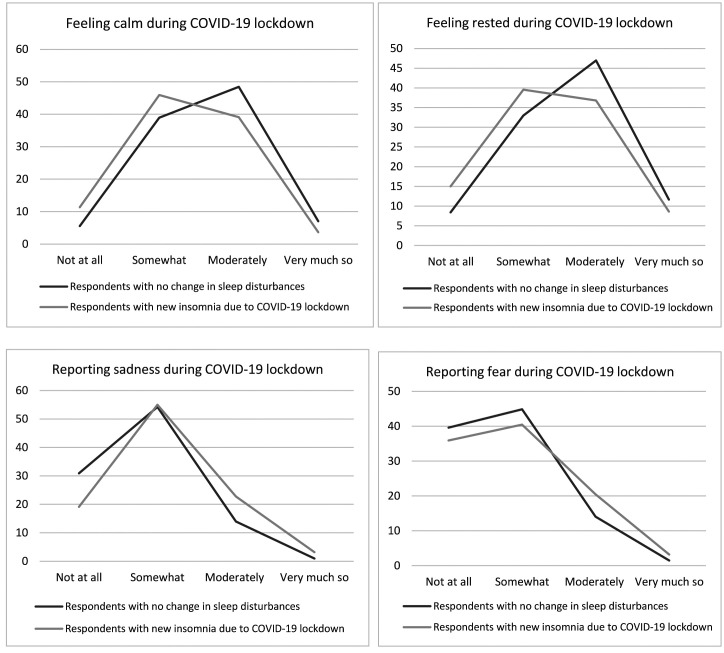
Mood assessment in respondents with self-reported first time insomnia during the coronavirus disease 2019 (COVID-19) lockdown compared with respondents with no sleep disturbances.

During the lockdown, respondents with newly reported insomnia more frequently reported sadness (χ^2^ = 24.011, P*<*0.001) and fear (χ^2^ = 9.093, *P* = 0.028), and less frequently reported feeling calm (χ^2^ = 17.724, P*<*0.001) and rested (χ^2^ = 15.862, *P* < 0.001) (Figure 3).

When logistic regression was performed for developing insomnia during the COVID-19 lockdown, an increased odds of developing insomnia during the lockdown was associated with younger age as well as with less common problems with night-time awakenings and less common problems falling asleep before the lockdown (Table 4).

**Table 4:** Logistic regression of self-reported first-time insomnia during the lockdown including age and sleep patterns before the lockdown as predictors*

	R^2^	B	P	Odds ratio	95% confidence interval for exponential value (B)	
Variables					lower	upper
**Age**	7.3%	-0.018	0.007	0.982	0.970	0.995
**Bedtime before lockdown**		-0.123	0.121	0.884	0.757	1.033
**Waketime before lockdown**		0.025	0.746	1.025	0.882	1.192
Problems with night-time awakenings before lockdown*		-0.569	0.048	0.566	0.322	0.996
Frequent problems falling asleep before lockdown*		-0.991	<0.001	0.371	0.230	0.599

*Problems with night-time awakenings before lockdown and frequent problems falling asleep before lockdown coded as 0 – no and 1 – yes.

## Discussion

In this study, respondents from the general population of Croatia experienced changes in sleeping patterns during the COVID-19 lockdown. Increased sleep latency and a significant delay in bedtime and waketime were reported, with the most pronounced delay in waketime and bedtime in respondents younger than 30 years. A pronounced delay in bedtime and waketime was also more evident in female respondents. An increased odds of self-reporting insomnia for the first time during the COVID-19 lockdown was associated with younger age and less common night-time awakenings, as well as with less common difficulties while falling asleep before the lockdown.

During the COVID-19 pandemic, complex demands are placed on sleep environments and social interactions, disfacilitating high-quality sleep (14). Poor sleep quality during the COVID-19 pandemic was reported worldwide (9,10,23-27). Additionally, sleep patterns shifted and sleep difficulties increased (30). A major finding of our study was a high prevalence of self-reported insomnia in the Croatian general population.

The onset of insomnia has been related to excessively stressful events (31). The vulnerability to insomnia is further promoted by a perceived intensity of stressfulness and lack of control by the respondent (32). Psychological stress during a lockdown results from social distancing with subsequent emotional behavior such as fear, depression, anxiety, and insomnia (8). Lockdown may be also perceived as a personalized traumatic event considering the reported increase in posttraumatic stress disorder (PTSD) symptoms during lockdown (4). PTSD-related sleep disturbances, such as increased awakenings and decreased slow wave sleep, are well-known (33,34) and frequently observed in insomnia sufferers (35). Furthermore, bedtime stress and worries have contributed to decreased sleep efficiency (36), especially during the COVID-19 outbreak (37). Furthermore, high levels of specific worries and loneliness related to COVID-19 have been associated with clinical levels of depression, anxiety, and PTSD symptoms (38). Having all this in mind, one might speculate that increased sleep latency reported in our study was a result of intrusive stress-related bedtime rumination during the COVID-19 lockdown.

Social distancing and overwhelming stress caused by long-term home confinement disturb a previously established positive balance between workload and home requirements, exercise and relaxation, as well as sleep and wakefulness (14). In our study, younger respondents and those with less common night-time awakenings and difficulties falling asleep before the COVID-19 lockdown had a greater risk of developing insomnia for the first time during the lockdown. This group might include those people who previously had well-established daytime and nighttime routines and who successfully balanced individual, social, and environmental demands. However, this balance and routines might have been upset by lockdown-related lifestyle and emotional disturbances, which increased the risk for self-reported insomnia. Furthermore, insomnia is associated with hyperarousal and overall negative mood, with more negative feelings in the evening (39). This agrees with the more frequent reports of sadness and fear of self-reported insomnia sufferers during the lockdown.

Another major finding of our study was a significant delay in bedtime and waketime during the COVID-19 lockdown, which was most pronounced in respondents under 30 years of age and in women. It remains to be resolved whether the observed shift was affected by the female parental responsibilities and work schedules.

Staying indoors during the day reduces the beneficial effects of natural daytime light exposure on sleep (40), and artificial light suppresses the regular circadian rhythm (40,41). The expanding use of electronic devices that emit blue-light, particularly before bedtime, delays sleep onset, increases sleep latency, alters melatonin secretion and synthesis, and increases alertness (40,41). Therefore, the bedtime shift and prolonged sleep latency observed in our study are not surprising given the restricted opportunities for leaving home during the lockdown and an overall increase in social media use and screen exposure, probably resulting from a need for enhanced social support during the pandemic (14,42,43). The delay in bedtime and waketime during the COVID-19 lockdown was followed by an increased total sleep time, because of a longer shift in waketime rather than bedtime, indicating a pre-existing sleep deprivation, as well as sleep debt. The shift in bedtime and waketime did not differ between the respondents from Zagreb and respondents from other parts of Croatia. However, respondents from Zagreb reported more frequent night-time awakenings and difficulties falling asleep. Sleep disturbances are a pervasive hallmark of natural disaster exposure, emerging in the form of frequent trauma-related disruptive night-time behaviors and decreased sleep quality (44).

There are several limitations to our study. The study enrolled only the respondents with internet access. Furthermore, the use of self-report data might have been a source of bias. Given the proportion of women represented (73.7%), as well as a large proportion of highly educated respondents (61.9%), our findings might be less representative of the general population. Women were more likely to voluntarily participate in the current study, and a similar trend was observed in our previous study (16). Furthermore, this study did not assess the prevalence of respondents who tested positive for COVID-19 in the general population. Moreover, during the lockdown, the respondents experienced the spring daylight-saving time transition, which is followed by an increase in sleep latency and sleep fragmentation (45). Still, we believe that the clock change impact was not as long-lasting and strong as the impact of the lockdown itself.

The lockdown burden on sleep needs to be better understood. Sleep hygiene education could serve a first-line lifestyle intervention for people in a lockdown experiencing sleep disruption. Further studies should aim at delivering these interventions to the vulnerable population groups recognized in the current study.
